# Deceased Donor Uterus Transplantation: A Narrative Review of the First 24 Published Cases

**DOI:** 10.3390/medicina60081348

**Published:** 2024-08-19

**Authors:** Basilio Pecorino, Giuseppe Scibilia, Martina Ferrara, Pierfrancesco Veroux, Benito Chiofalo, Paolo Scollo

**Affiliations:** 1 Obstetrics and Gynecology Umberto I Hospital, Kore University of Enna, 94100 Enna, Italy; basilio.pecorino@unikore.it; 2 Obstetrics and Gynecology Giovanni Paolo II Hospital, 97100 Ragusa, Italy; g.scibilia@libero.it; 3 Obstetrics and Gynecology Cannizzaro Hospital, 95126 Catania, Italy; martiferra@hotmail.it; 4 Vascular Surgery and Organ Transplant Unit, Department of General Surgery and Medical-Surgical Specialties, University Hospital of Catania, 95124 Catania, Italy; pveroux@unict.it; 5 Obstetrics and Gynecology Cannizzaro Hospital, Kore University of Enna, 95126 Catania, Italy; paolo.scollo@unikore.it

**Keywords:** uterus transplantation, deceased donor, living donor, birth rate, AUFI

## Abstract

Uterus transplantation is the surgical treatment for absolute uterine factor infertility (AUFI), a congenital or acquired condition characterized by the absence of a uterus. More than 80 transplants have been performed worldwide, resulting in more than 30 live births, originating both from living and deceased donors. The collection of published articles on deceased donor uterus transplantations was performed in PubMed and SCOPUS by searching for the terms “Uterus transplantation” AND “deceased donor”; from the 107 articles obtained, only case reports and systematic reviews of deceased donor uterus transplantations and the resulting live births were considered for the present manuscript. The extracted data included the date of surgery (year), country, recipient (age and cause of AUFI) and donor (age and parity) details, outcome of recipient surgery (hysterectomy), and live births (date and gestational age). The search of peer-reviewed publications showed 24 deceased donor uterus transplantations and 12 live births (a birth rate of 66%) with a 25% occurrence of graft loss during follow-up (6 of 24). Among this series, twelve transplants were performed in the USA (seven births), five in the Czech Republic (one birth), three in Italy (one birth), two in Turkey (two births), and two in Brazil (one birth). The median recipient age was 29.8 years (range 21–36), while the median donor age was 36.1 years (range 20–57). Of 24 recipients, 100% were affected by MRKH (Mayer-Rokitanski-Kuster-Hauser) syndrome. Two live births were reported from nulliparous donors. Deceased donor uterus transplantation birth rates are very similar to the living donor rates reported in the literature, but ethical implications could be less important in the first group. It is necessary to register every case in the International Registry for Uterus Transplantation in order to perform a systematic review and comparison with living donor rates.

## 1. Introduction

Absolute uterine factor infertility (AUFI) is the congenital or acquired absence of a uterus [1]. The most frequent cause of AUFI is Mayer–Rokitansky–Küster–Hauser (MRKH) syndrome, a condition characterized by varying degrees of vaginal and uterus agenesia. Non-surgical options for these patients include surrogacy or adoption, but both are highly dependent upon the legal jurisdiction in the country of residence [2]. Surrogacy is banned in many countries and its application is complicated by various ethical issues around gender, labor, payment, exploitation, and inequality. Adoption also has ethical problems, and its application can be difficult due to bureaucratic factors. For these reasons, the global demand for uterus transplantation (UTx) is constantly increasing, providing the opportunity to experience pregnancy and to be a gestational mother.

Uterus transplantation (UTx) is a surgical treatment for AUFI and it is a rapidly expanding technique all over the world.

To date, more than 80 transplants have taken place, from both living and deceased donors, resulting in more than 30 births [3]. The first UTx was performed in Saudi Arabia in 2000 from a deceased donor, but that attempt failed [4]. The first live birth from UTx was in Sweden in 2014 [5], from a living donor (LD), while the first live birth after UTx from a deceased donor was in Brazil in 2017 [6].

Both techniques have demonstrated successful outcomes in terms of uterus function and pregnancy, and their advantages and disadvantages have been extensively debated [7].

With living donors, there is a possibility of extensively evaluating their obstetric, medical, and family history, as well as of testing pre-transplant; surgery can be electively planned for the donor and the recipient, with a clear process for informed consent; the donor experiences a sense of pleasure from helping another person, whether they are familiar to them or not. Unfortunately, there are several risks during surgery, such as bleeding, infection, pain, ureteral and bladder injuries, and ovarian dysfunction; also, since the age of donor is often older, consequentially, the possible sense of guilt of the recipient must be considered. 

With deceased donors, there are advantages, such as the ability to obtain longer vascular grafts and a longer vaginal cuff, which improve, respectively, graft success and vagino–vaginal anastomosis. The disadvantages include the limited availability of organs, a longer cold ischemia time, and the inability to plan surgeries and extensively test the donor. 

Without considering the problems arising during surgery, given that the outcomes are similar, deceased donors could be the preferred donors for ethical reasons. Since successful outcomes of deceased donor UTx were reported, trials have been ongoing in several countries (the USA, Brazil, the Czech Republic, and Italy) and the number of procedures performed will increase in the next few years. The aim of this manuscript is to review the literature about published cases of deceased donor UTxs and live births after this procedure. 

## 2. Materials and Methods

The last report of the Registry of the International Society of Uterus Transplantation was published in 2023 [8]. Unfortunately, the number of registered procedures is lower than the real number of UTxs performed, so, when researching for an exhaustive review of the literature, we must consider other sources.

The selection of published articles on deceased donor UTxs was performed in PubMed and SCOPUS, by searching for the terms “Uterus transplantation” AND “deceased donor”. The research was performed according to the PRISMA statement (see the Supplementary Materials) and registered in PROSPERO (ID: 557675). Of the 107 articles obtained, only case reports and systematic reviews of deceased donor UTxs and their subsequent live births were considered for the present manuscript.

The extracted data included the date of surgery (year), country, recipient (age and cause of AUFI) and donor (age and parity) details, outcome of recipient surgery (hysterectomy), and live births (date and gestational age).

A review of the literature was also performed to highlight the state of the art of worldwide deceased donor UTx activity, the differences between living and deceased donations, the surgical technique for uterus retrieval from deceased donors, and vaginal stenosis.

## 3. Results

### 3.1. Worldwide Deceased Donor UTx Activity

The search for publications showed 24 deceased donor UTxs and 12 live births (a birth rate of 50%), with a 25% occurrence of graft loss during follow-up (6 of 24). The nations involved in programs for deceased donor UTx are Turkey, the USA (specifically Cleveland, Dallas, and Pennsylvania), Brazil, the Czech Republic, and Italy. Table 1 summarizes the case series by country.

The review of the literature showed two transplantations with two live births in Turkey, with the first UTx performed in 2011 [9] and the second one in 2021 [10]. The first live birth after deceased donor UTx in Turkey occurred 9 years after the transplantation, and this was the first uterus transplant that successfully led to a live birth in the world [11]. Both women received immunosuppressive therapy using tacrolimus, azathioprine, and prednisolone, and no signs of clinical rejection were registered. The pregnancies were complicated by preterm premature rupture of membrane and preterm labor, and cesarean sections were performed, respectively, at 28 and 29 weeks of gestational age [12]. 

In the USA, the US Uterus Transplant Consortium is formed of three centers: Baylor University Medical Center in Dallas, Cleveland Clinic in Cleveland, and the University of Pennsylvania in Philadelphia. The first transplant was performed at Cleveland Clinic in 2016 [13], but the graft failed 12 days after the transplant. The first live birth after UTx in the USA was achieved at Baylor Center in 2018 [14]. The American UTx experience is the largest case series in the world, with 33 transplants, of which 12 are from deceased donors [15], and seven live births from these patients. 

The Czech Republic team published the results of their first ten cases of UTx, of which five were deceased donor UTx [16]. Among deceased donor UTx patients, one live birth was registered as the first live birth from deceased donor UTx in Europe and the first live birth from a nulliparous donor worldwide [17].

Another two deceased donor UTxs were registered in Brazil, of which the first one was the first live birth from a deceased donor in the world [6].

The Italian team performed much research about the issues related to oocyte retrieval in patients affected by Mayer–Rokitansky–Küster–Hauser (MRKH) syndrome, specifically on ectopic ovaries [18] and psychological disorders in MRKH patients [19]. In Italy, three deceased donor UTxs were performed and the first resultant live birth occurred in 2022 [20], presented as the first case in the world obtained from cryopreserved oocytes and not embryos, for ethical reasons. 

Table 2 summarizes case-by-case reports of DD UTxs and live births.

The median recipient age was 29,8 years (range 21–36), while the median donor age was 36,1 (range 20–57). Of 24 recipients, 100% were affected by MRKH syndrome. Five donors were nulliparous, one patient had one child, and five patients had two or more children. 

### 3.2. Differences between Living (LD) and Deceased Donation (DD)

The international debate about the type of donor (living or deceased) is still open, as LD UTx is a very invasive surgery for the donor, and DD UTx is preferred for ethical reasons but is limited by organ availability. The ethical issues of DD UTx include consent for donation and the procurement of the uterus in relation to other vital organs [25]. Uterus procurement requires explicit consent from the deceased donor or their family. The retrieval of the uterus during multi-organ procurements is usually performed at the end of the procedure, after the vital organs, but this practice may significantly increase the ischemia time of the uterus. Conversely, the ethical issues regarding LD UTx are more complex as they include the donor, the recipient, and the child; for example, pressure on the donor to donate, complications requiring surgical treatment for the donor, the legal rights to the donated uterus and the eventual children, and the expectation of a relationship between the child and the donor.

The surgery for uterus retrieval from a LD is a complex procedure, burdened by several potential complications including bowel injuries, uretero–vaginal fistula, ureteral or bladder injuries, and nerve damage beyond those common risks of surgery through infections, bleeding, and thrombosis [26]. More than 10% of donors need further surgery to resolve the complications of uterus retrieval [2]. Another important risk for young donors is early menopause due to ovarian failure stemming from ovarian veins being utilized for vascular anastomosis [27].

The risks for the donor are not only physical but also psychological [28]. Firstly, hysterectomy can lead to a perceived loss of femininity and decreased sexual libido and satisfaction, leading to psychological distress in the donor. Furthermore, anxiety and depression in the recipient could arise from an early failure of the graft or from not becoming pregnant [29]. The recipients are, in most cases, affected by MRKH syndrome, a condition associated with hypochondria, depression, hysteria, physical dysfunctions, lassitude-malaise, and somatic complaints [19]. In LD UTx, the recipient may also feel fear about the donor’s health and potential serious complications that may occur after surgery. 

The real advantage of LD is the evaluation of the donor [30]. In fact, there is an opportunity for a deep evaluation of the patient and the uterus before donation, increasing the possibility of success of transplantation and pregnancy. The donor’s obstetric and gynecological history, including anamnesis of preterm birth or other severe conditions such as pre-eclampsia and post-partum hemorrhage, can lead to their exclusion. In addition, as the donor is often a close relative of the recipient (e.g., their mother or sister), there is a higher overlap of HLA antigens, which determine a good immune system tolerance. In case the donor is of menopausal age, it is possible to prescribe hormone replacement therapy to stimulate endometrial growth before transplantation [31].

The procurement of a uterus from a deceased donor is a faster procedure than from a living donor. In fact, the surgery does not lead to any damage, since the deceased donor is a brain-dead subject. The duration of DD surgery was reportedly about 60–90 min in the American trial [32] and about 120 min for the Turkish team [33]. 

There is great variability in the length of time taken for LD procurement reported in the literature, ranging from 6–8 h [34] to 10–13 h [35]. The duration has progressively reduced from about 10–13 h, in the first Swedish trial [36], to 6 h, during a Chinese robotic [37] procurement, but it remains significantly longer than with a DD.

In DD uterus retrieval, it is possible to obtain longer vascular and ligament pedicles in order to ensure improved function of vascular anastomosis and the support system and to increase the success rate by reducing thrombosis and rejection risks [7].

A disadvantage is that DD surgery has to be performed quickly, since brain-dead subject availability cannot be predicted; the retrieval must be rapid. The stress of surgeons, due to fatigue and the work often being performed at night, leads to an increased rate of complications [38].

Graft failure is an event that requires the removal of the uterus before embryo transfer [39]. The graft failure rate reported in the most recent review was 21% for LD and 36% for DD [25], but a comparison between the two is difficult, given the low number of DD UTx procedures. Based on our review, the graft failure rate in DD is 25% (6/24), approximately the same as LD. 

DD graft can be affected by negative effect of systemic inflammation and ischemia, “warm” inside the body and “cold” during the storage on ice, determining histologic and metabolic damages, eventually empowered in the phase of reperfusion and responsible to acute or chronic graft failure [38]. The cold ischemia time is longer in DD than in LD, but the uterus’ resistance to ischemic damage is high, as demonstrated in several studies [27], where durations greater than 9 h led to live birth [7].

Live birth is the principal aim of uterus transplantation. According to the most recent review comparing LD and DD uterus transplantation [25], the live birth rates are, respectively, 63% and 71 %. Our review reported a live birth rate of 66% (12/18), which is very similar to LD data.

### 3.3. Surgical Technique of Uterus Retrieval from Deceased Donor

Uterus retrieval typically takes place after the procurement of vital organs, although there are some variations reported in the literature [35]. A possible choice is to prepare the organs at the beginning of multi-organ procurement, and then to perform the dissection of the pedicles last. Uterus procurement from a DD, when performed using the standardized procedure, should not impact the excision of vital organs; it is performed, in several steps, during multi-organ retrieval from a deceased donor with confirmed brain death. All of the cases included in this review (24 of 24, 100%) involved traditional open surgery (longitudinal incision). The length of time taken for DD uterus retrieval was, in most cases, less than 3 h.

For both DD and LD uterus retrieval, obtaining a regular venous drainage is critical, as one of the most common reasons for graft loss is venous thrombosis [39].

The American team reported that, during multi-organ procurement, the uterus is prepared early and the removal occurs either prior to the retrieval of the other solid organs, or last [15]. The surgical technique was described by Testa et al. [24]. The round ligaments are sectioned as close as possible to the pelvic wall, the anterior peritoneum is incised, and the bladder is separated from the uterus and vagina. The external iliac arteries are dissected to the level of the internal iliac artery, and all branches of the latter except the uterine artery are ligated. The distal branches of the internal iliac vein are also ligated, preserving the uterine vein. The utero-sacral ligaments are transected and then the vagina is sectioned, 2–3 cm below the cervix. The ovarian vessels are ligated proximal to the ovaries, while the ovaries and fallopian tubes are subsequently removed on the back table. The internal iliac vein is clamped and the uterus is flushed through bilateral uterine arteries on the back table using University of Wisconsin solution. 

Fronek et al. [40] described, in particular, the phase of retrieval. The first step includes a gynecological ultrasound, the second step is a macroscopic assessment of the uterus, the third step involves the preparation of the uterus and vascular pedicles, and the last step comprises the perfusion, removal, and assessment of the quality of the graft. Regarding the surgical preparation of the uterus, the round ligaments are sectioned at the pelvic wall, ovarian veins are prepared bilaterally, the uterus is dissected from the bladder, and the internal iliac artery, uterine artery, internal iliac vein, and uterine vein are prepared and marked. After perfusion, all organs selected for procurement are successively excised, and the uterus, being a non-lifesaving organ, is removed last. 

Ozkan [10] described a retrieval process of 120 min and the removal of the graft before any of the other organs, in order to obtain a longer common iliac artery and veins from the donor.

The Italian procedure (our group, not yet published data) of uterus retrieval is based on the same surgical times as abdominal open radical hysterectomy, considering the project leader’s experience in gynecological oncology. The round ligament is transected as close as possible to the pelvic wall, and then, the pelvic peritoneum is incised and the retroperitoneum is prepared, to develop paravesical and pararectal spaces. The uterine artery and the superficial vein are transected as close as possible to their origin; in some cases, the pedicle also includes a brief segment of the iliac artery and vein.

### 3.4. Vaginal Stenosis

Vaginal stenosis is a complication of uterus transplantation characterized by a stricture in the vagino–vaginal anastomosis, between the recipient vagina and the vaginal cuff of the donor. The occurrence of vaginal stenosis is likely underestimated in the literature, but a report of the first 5 years of UTx activity in the USA reported an incidence of 72% (18 of 33) [15], and the interim results of the first 10 cases in the Czech Republic reported an incidence of 63% [16]. In the UTx Italian project, the incidence of vaginal stricture was 66% (2 of 3) (not yet published, authors of the present review). Vaginal stenosis represents a big limitation in follow-up post-transplantation, because it makes rejection monitoring difficult and it delays embryo transfer; it also limits the outflow of menstrual blood and sexual satisfaction [21]. In the Czech series, six of seven patients underwent surgery for a laparoscopic neovagina using the Vecchietti procedure; stenosis was corrected through surgical dilatation and stent placement, but the type of device was not described. One case developed vesical–vaginal fistula after surgical treatment involving resection of stenosis with a diathermy knife. The American authors reported surgical correction with nonsurgical dilation in half of the cases and with surgical dilation in the other half.

**Figure 1:**
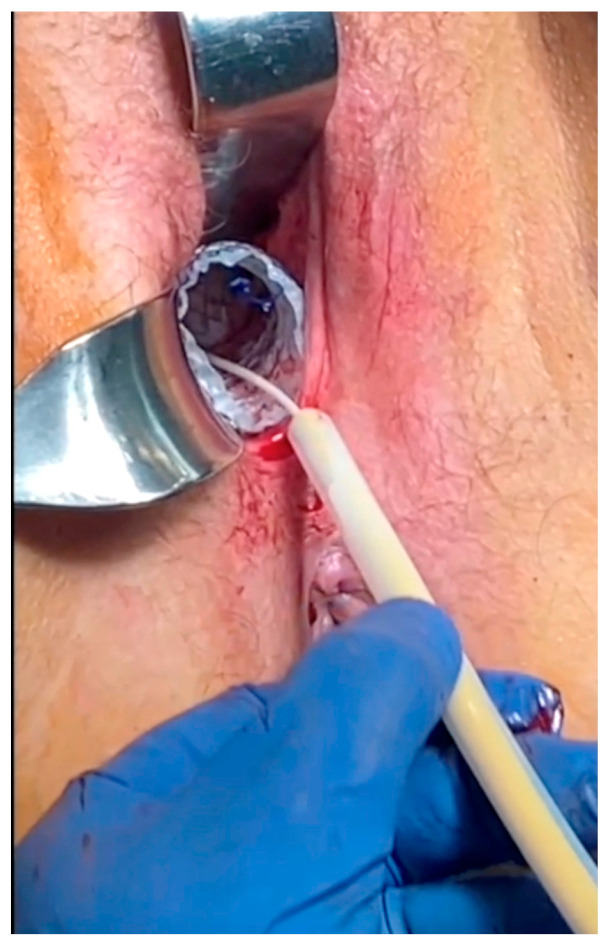
Vaginal off-label stent placement.

The Italian UTx team performed successful surgical correction of stenosis by the positioning of an off-label stent (data not yet published) in both cases (Figure 1). 

Rehmer described a new technique of vaginal anastomosis in order to minimize the risk of stenosis [41], but the procedure was not standardized and there is not an international consensus about this. 

The most recent manuscript, by Johannesson et al. [42], standardizes the classification and treatment of vaginal stricture in UTx patients. The severity of stenosis is graded using measurements of stricture: 2–3 cm (grade 1), 1–2 cm (grade 2), and <1 cm (grade 3). Low and moderate stenosis were treated using self-dilatation in more than half of cases, while severe stenosis required surgical treatment.

## 4. Discussion

Uterus transplantation is a valid option for patients affected by AUFI, as an alternative to adoption and surrogacy. Today, more than 80 transplants have been performed with over 40 live births, both from living and deceased donors. Approximately 66% of UTx procedures are from living donors [43]. Brannstrom et al. [44] reported a surgical success rate of 78% in LD and 64 % in DD, with similar (18% vs. 19 %) complications rates requiring invasive or radiological intervention in the recipients. A recent review of the first 5 years of UTx activity in the USA [15] reported a 1-year graft survival rate of 74% and 75% after LD and DD UTx, respectively, which constitute highly comparable data.

The present manuscript considers the worldwide UTx deceased donor activity by reviewing previously published cases. Unfortunately, this is mostly a narrative review, because detailed data about every case—for example, the duration of surgery and warm and cold ischemia time—are not available in the already published manuscripts. Nations that have published results of large series (for example, the USA) do not report detailed data about every single case, so it is impossible to perform a systematic review of the literature.

The International Society of Uterus Transplantation was founded in 2017, and its activity is based on an online registry, with prospective data including UTx procedures and any relevant data until 3 months after transplant hysterectomies [8]. The registry includes data about donors, recipients, surgeries (donor hysterectomy and transplantation), immunosuppressive therapies, rejections, assisted reproduction, pregnancies, live births, and transplant hysterectomies. Postoperative complications within 30 days are also registered and graded based on the Clavien–Dindo (CD) classification system. The registry is a useful tool for systematically reporting all the data about every single transplant, but, unfortunately, the first manuscript of the International Society published in January 2023 contains only information about 45 UTx procedures, 35 LD and 10 DD. The median ages of the deceased donors and recipients were 38,5 years and 29 years, respectively. The authors reported a total of 19 live births from 16 recipients (14 after LD UTx and 2 after DD UTx).

Considering the limited availability of data, comparisons between living donor and deceased donor UTx are difficult to make. According to the most recent review comparing LD and DD uterus transplantation [25], the live birth rates are, respectively, 63% and 71%. Our review reported a live birth rate of 66% (12/18), which is very similar to the LD data.

Another limit that must be considered is the low number of DD UTxs performed, considering that the availability of organs is low. In the future, uterus retrieval for UTx could be less invasive for living donors because alternative surgical techniques, such as robotic surgery, are progressively increasing.

## 5. Conclusions

In conclusion, the outcomes of UTx in terms of graft success, live births, and complication rates between LD and DD are comparable, but they are limited by the low availability of systematic reviews. Further research is necessary to increase the number of DD UTxs and to obtain a large series to compare with LD UTxs. The registration of all cases in the International Registry of International Society of Uterus Transplantation is highly recommended.

## Figures and Tables

**Table 1:** Deceased donor UTx, live births, and graft failures.

Country	N. of DD UTxs	N. of Live Births	Graft Failures
Turkey	2	2	0
USA	12	7	3
Brazil	2	1	0
Czech Republic	5	1	2
Italy	3	1	1
**Total**	**24**	**12**	**25%**

**Table 2:** Summary of DD UTx case reports around the world.

N.	Year	Country	Recipient Age	Donor Age	Cause of AUFI	Parity of Donor	Outcome	Live Birth (n.), Gestational Age (w + d), Obstetrics Complications
1	2011	Turkey [9,11]	21	22	MRKH syndrome	0	Cesarean hysterectomy	1, 28 + 0, IUGR, pre-eclampsia
2	2016	USA (Cleveland) [13]	29	30	MRKH syndrome	NR	Hysterectomy day 12	NA
3	2016	USA (Baylor) [15]	36	33	MRKH syndrome	3	Cesarean hysterectomy	1, 37 + 6
4	2016	Czech Republic [21]	30	57	MRKH syndrome	1	Hysterectomy day 7	0
5	2016	Czech Republic [22]	26	24	MRKH syndrome	0	In situ	0
6	2016	Brazil [6]	32	45	MRKH syndrome	3	Cesarean hysterectomy	1, 35 + 3, pyelonephritis
7	2017	Czech Republic [17]	25	20	MRKH syndrome	0	Cesarean hysterectomy, vaginal stenosis	1, 34 + 6, diabetes mellitus
8	2017	Czech Republic [23]	33	56	MRKH syndrome	1	Hysterectomy month 7, vaginal stenosis	0
9	2017	USA (Baylor) [24]	29	44	MRKH syndrome	NR	Graft loss	0
10	2017	USA (Cleveland) [14]	35	24	MRKH syndrome	NR	Cesarean hysterectomy	1, 34 + 1, placenta accreta, impaired renal function
11	2018	Czech Republic [16]	29	45	MRKH syndrome	NR	In situ, vaginal stenosis	0
12	2018	USA (Penn) [15]	33	NR	MRKH syndrome	NR	NR	1
13–17	2019	USA [15]	NR	NR	NR	NR	NR	3
18	2019	USA (Cleveland)	31	NR	NR	NR	NR	1
19	2020	Italy [20]	27	37	MRKH syndrome	2	In situ, vaginal stenosis	1
20	2020	USA [15]	NR	NR	NR	NR	NR	NR
21	2021	Turkey [12]	32	37	MRKH syndrome	3	In situ	1, 29 + 0, PPROM
22	2022	Italy (in press)	33	43	MRKH syndrome	0	In situ, vaginal stenosis	0
23	2023	Italy (in press)	27	25	MRKH syndrome	0	Graft rejection	0
24	NR	Brazil [12]	NR	NR	NR	NR	NR	0

Legend: NR = not reported; NA = not applicable; MRKH = Mayer–Rokitansky–Küster–Hauser; IUGR = intrauterine growth restriction; PPROM = preterm premature rupture of membranes.

## Supplementary Materials

The following supporting information can be downloaded at: https://www.mdpi.com/article/10.3390/medicina60081348/s1, Figure S1: PRISMA flow diagram.
