# Supplementary figures and images for: Deceased Donor Uterus Transplantation: A Narrative Review of the First 24 Published Cases
Source: Medicina (Kaunas). 2024 Aug 19;60(8):1348. doi: 10.3390/medicina60081348 (PMC11356378; doi:10.3390/medicina60081348)

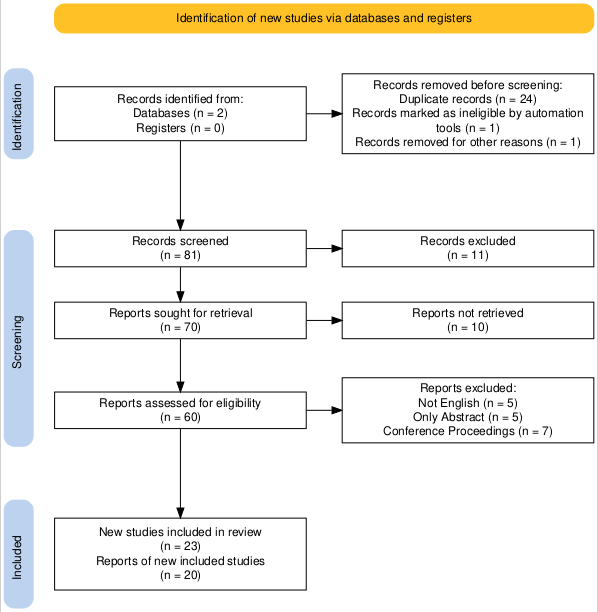

Supplement: Supplementary file 1 [file medicina-60-01348-s001.zip › medicina-3070828-Figure S1.jpg]
